# Supplementary material for: Perimount MAGNA Ease vs. INSPIRIS Resilia Valve: A PS-Matched Analysis of the Hemodynamic Performances in Patients below 70 Years of Age
Source: J Clin Med. 2023 Mar 6;12(5):2077. doi: 10.3390/jcm12052077 (PMC10004583; doi:10.3390/jcm12052077)
Supplement: Supplementary file 1 [file jcm-12-02077-s001.zip › jcm-2197397-supplementary.pdf]

Supplemental Tables

Supplemental Table S1. Overall population: patient characteristics

	Total	Perimount Magna Ease	Inspiris Resilia	p-value Cohens d
	n/N (%) or Mean ± SD /Median (IQR) n=430	n/N (%) or Mean ± SD /Median (IQR) n=238	n/N (%) or Mean ± SD /Median (IQR) n=192	
Age, years	58.0 ± 9.6<br>60 (53;66)	59.4 ± 10.0<br>63 (55.8;67)	56.3 ± 8.7<br>57.5 (51.3;63)	*<0.001-*<br>*0.328*
Female gender	96 (22.3)	56 (23.5)	40 (20.8)	0.505
BMI, kg/m^2	26.9 ± 4.4	26.9 ± 4.0	26.9 ± 4.9	0.433
Diabetes mellitus	49 (11.4)	44 (18.5)	5 (2.6)	*<0.001*
COPD	43 (10.0)	29 (12.2)	14 (7.3)	0.093
Creatinine, mg/dl	1.0 ± 1.0<br>417	1.0 ± 1.0<br>225	1.0 ± 1.1<br>192	0.578
Dialysis	5 (1.2)	5 (2.1)	0 (0.0)	0.068
NYHA				
I	88 (20.5)	7 (2.9)	81 (42.2)	
II	227 (52.8)	172 (72.3)	55 (28.6)	*<0.001*
III	58 (13.5)	17 (7.1)	41 (21.4)	
IV	57 (13.3)	42 (17.6)	15 (7.8)	
NYHA III/IV	115 (26.7)	59 (24.8)	56 (29.2)	0.308
EuroScore II, %	2.6 ± 2.7	2.6 ± 2.7	2.6 ± 2.7	0.513
Hypertension	247 (57.4)	155 (65.1)	92 (47.9)	*<0.001*
Dyslipidaemia	144 (33.5)	97 (40.8)	47 (24.5)	*<0.001*
Smoker				
never	266 (61.9)	161 (67.6)	105 (54.7)	
active	73 (17.0)	19 (8.0)	54 (28.1)	*<0.001*
former	91 (21.2)	58 (24.4)	33 (17.2)	
Peripheral vascular disease	56 (13.0)	43 (18.1)	13 (6.8)	*<0.001*
History of stroke	17 (4.0)	12 (5.0)	5 (2.6)	0.197
Atrial fibrillation				
Paroxysmal	23 (5.3)	19 (8.0)	4 (2.1)	*0.021*
Persistent	34 (7.9)	20 (8.4)	14 (7.3)	
Pacemaker	9 (2.1)	7 (2.9)	2 (1.0)	0.310
Prev. MI	26 (6.0)	20 (8.4)	6 (3.1)	*0.022*
Prior PCI	26 (6.0)	17 (7.1)	9 (4.7)	0.288
Prior cardiac surgery	42 (9.8)	21 (8.8)	21 (10.9)	0.463
Coronary artery disease	86 (20.0)	53 (22.3)	33 (17.2)	0.190
Endocarditis	30 (7.0)	13 (5.5)	17 (8.9)	0.170
Ejection fraction, %	57.4 ± 10.4	58.2 ± 9.6	56.5 ± 11.3	0.687
**Preoperative Echo**				
Bicuspid valve	151/385 (39.2)	54/224 (24.1)	97/161 (60.2)	*<0.001*
End-diastolic volume, ml	153.0 ± 61.1<br>142.5 (115.0;180.0)<br>217	158.4 ± 61.5<br>145.0 (117.5; 187.5)<br>57	151.0 ± 61.0<br>142.0 (112.8;175.0)<br>160	0.438
End-diastolic diameter, mm	57.1 ± 15.5<br>112	56.2 ± 10.0<br>63	58.3 ± 20.7<br>49	0.718

Interventricular septum, mm	13.4 ± 6.1<br>209	14.1 ± 8.8<br>92	12.8 ± 2.2<br>117	0.221
Peak gradient, mmHg	65.5 ± 25.4<br>186	69.9 ± 21.7<br>74	62.7 ± 27.3<br>112	0.058
Mean gradient, mmHg	45.8 ± 15.7<br>193	44.3 ± 14.9<br>76	41.8 ± 16.2<br>117	0.252
Vmax, cm/sec	3.8 ± 0.9<br>48	4.3 ± 0.6<br>8	3.7 ± 0.9<br>40	0.565
AVA, cm^2	0.76 ± 0.53<br>106	0.38 ± 0.50<br>26	0.88 ± 0.48<br>80	*0.003*<br>1.031
Pulmonary artery pressure systolic, mmHg	37.2 ± 13.1<br>113	37.9 ± 10.6<br>35	36.9 ± 14.1<br>78	0.318
Aortic regurgitation				
No	155/414 (37.4)	118/238 (49.6)	37/176 (21.0)	
Mild	78/414 (18.8)	41/238 (17.2)	37/176 (21.0)	*<0.001*
Moderate	66/414 (15.6)	23/238 (9.7)	43/176 (24.4)	
Severe	115/414 (27.8)	56/238 (23.5)	59/176 (33.5)	
Moderate/severe	181/414 (43.7)	79/238 (33.2)	102/176 (58.0)	*<0.001*
Mitral regurgitation				
No	112/264 (42.4)	70/108 (64.8)	42/156 (26.9)	
Mild	111/264 (42.0)	24/108 (22.2)	87/156 (55.8)	*<0.001*
Moderate	30/264 (11.4)	12/108 (11.1)	18/156 (11.5)	
Severe	11/264 (4.2)	2/108 (1.9)	9/156 (5.8)	
Moderate/severe	41/264 (15.5)	14/108 (13.0)	27/156 (17.3)	0.338
Tricuspid regurgitation				
No	103/173 (59.5)	46/50 (92.0)	57/123 (46.3)	
Mild	56/173 (32.4)	2/50 (4.0)	54/123 (43.9)	*<0.001*
Moderate	9/173 (5.2)	1/50 (2.0)	8/123 (6.5)	
Severe	4/173 (3.3)	1/50 (2.0)	4/123 (3.3)	
Moderate/severe	14/173 (8.1)	2/50 (4.0)	12/123 (9.8)	0.356

Legend: AVA, aortic valve area; BMI, body mass index; COPD, chronic obstructive pulmonary disease; MI, myocardial infarction; NYHA, New York Heart Association; PCI, percutaneous coronary intervention

Supplemental Table S2. Overall population: periprocedural outcome

	Total	Perimount Magna Ease	Inspiris Resilia	p-value Cohens d
	n/N (%) or Mean ± SD n=430	n/N (%) or Mean ± SD n=238	n/N (%) or Mean ± SD n=192	
Aortic prosthesis size				
19mm	24 (5.6)	9 (3.8)	15 (7.8)	0.388
21mm	67 (15.6)	36 (15.1)	31 (16.1)	
23mm	141 (32.8)	75 (31.5)	66 (34.4)	
25mm	129 (30.0)	78 (32.7)	51 (26.6)	
27mm	60 (14.0)	34 (14.3)	26 (13.5)	
29mm	9 (2.1)	6 (2.5)	3 (1.6)	
Isolated AVR	217 (50.5)	126 (52.9)	91 (47.4)	0.253
Concomitant procedure				
Mitral valve surgery	31 (7.2)	19 (8.0)	12 (6.3)	0.490
Mitral valve replacement	10 (2.3)	7 (3)	3 (1.6)	
Mitral valve repair	21(4.8)	12 (5)	9 (4.7)	
Root replacement	23 (5.3)	10 (4.2)	13 (6.8)	0.239
Ascending aorta replacement	82 (19.1)	41 (17.2)	41 (21.4)	0.279
CABG	51 (11.9)	33 (13.9)	18 (9.4)	0.178
other	26 (6.0)	9 (3.8)	17 (8.9)	*0.028*
Cardiopulmonary bypass time, min	106.1 ± 40.3	106.9 ± 37.7	105.2 ± 43.0	0.300
Cross clamp time, min	83.8 ± 30.5	82.5 ± 30.7	85.2 ± 30.3	0.271
Procedural mortality	0 (0)	0 (0)	0 (0)	n.a.
Post-operative stay, days	8.5 ± 6.8	8.8 ± 6.9	8.2 ± 6.7	0.074
ICU stay, days	2.5 ± 6.3	2.7 ± 6.0	2.3 ± 6.7	*<0.001* -0.063
Periprocedural mortality	1 (0.2)	1 (0.4)	0 (0)	1.000
Dialysis	5/393 (1.3)	5/201 (2.5)	0/192 (0)	0.061
New onset atrial fibrillation				
Paroxysmal	103 (24.0)	58 (24.4)	45 (23.4)	0.760
Persistent	4 (0.9)	3 (1.3)	1 (0.5)	
PM implantation	12 (2.8)	8(3.4)	4(2.1)	0.7
Neurological events				
Type 1	2 (0.5)	1(0.4)	1 (0.5)	0.6
Type 2	3(0.2)	1 (0.4)	2(1.0)	
Type 3	3(0.2)	2(0.8)	1(0.5)	
Bleeding requiring surgical revision	16 (3.7)	12 (5)	4 (2.1)	2.5
NYHA class at discharge				
I	374/403 (92.8)	219/237 (92.4)	155/166 (93.4)	0.274
II	25/403 (6.2)	17/237 (7.2)	8/166 (4.8)	
III	0	0	0	
IV	4/403 (1.0)	1/237 (0.4)	3/166 (1.8)	
NYHA III/IV	4/403 (1.0)	1/237 (0.4)	3/166 (1.8)	0.310
Medical treatment at discharge				
Vitamin k-antagonists/NOAC	380/390 (97.4)	197/198 (99.5)	183/192 (95.3)	*0.010*

ASA	150/389 (38.6)	56/197 (28.4)	94/192 (49.0)	*<0.001*
Dual antiplatelet therapy	2/389 (0.5)	2/197 (1.0)	0/192 (0)	0.499

Legend: AVR, aortic valve replacement; CABG, coronary artery bypass graft

Supplemental Table S3. PS matched population: periprocedural outcome

	Total	Perimount Magna Ease	Inspiris Resilia	p-value Cohens d
	n/N (%) or Mean ± SD n=244	n/N (%) or Mean ± SD n=122	n/N (%) or Mean ± SD n=122	
Aortic prosthesis size				
19mm	15 (6.1)	3 (2.5)	12 (9.8)	
21mm	40 (16.4)	17 (13.9)	23 (18.9)	
23mm	71 (29.1)	29 (23.8)	42 (34.4)	*0.007*
25mm	73 (29.9)	44 (36.1)	29 (23.8)	
27mm	39 (16.0)	25 (20.5)	14 (11.5)	
29mm	6 (2.5)	4 (3.3)	2 (1.6)	
Isolated AVR	112 (45.9)	55 (45.1)	57 (46.7)	0.797
Concomitant procedure				
Mitral valve surgery	22 (9.0)	13 (10.7)	9 (7.4)	0.371
Root replacement	14 (5.7)	7 (5.7)	7 (5.7)	1.000
Ascending aorta replacement	53 (51.7)	30 (24.6)	23 (18.9)	0.277
CABG	25 (10.2)	10 (8.2)	15 (12.3)	0.291
other	18 (7.4)	7 (5.7)	11 (9.0)	0.327
Cardiopulmonary bypass time, min	106.9 ± 40.7	114.4 ± 40.2	100.5 ± 40.3	0.003
Cross clamp time, min	86.0 ± 32.3	88.5 ± 33.6	83.9 ± 31.1	0.332
Procedural mortality	0 (0)	0 (0)	0 (0)	n.a.
Post-operative stay, days	8.7 ± 7.3	9.2 ± 8.1	8.3 ± 6.4	0.195
ICU stay, days	2.3 ± 5.3	2.4 ± 4.2	2.3 ± 6.1	*<0.001* -0.019
Periprocedural mortality	0 (0)	0 (0)	0 (0)	n.a.
Dialysis	4/233 (1.7)	4/111 (3.6)	0/122 (0)	*0.050*
New onset atrial fibrillation				
Paroxysmal	57 (23.4)	27 (22.1)	30 (24.6)	0.881
Persistent	2 (0.8)	1 (0.8)	1 (0.8)	
PM implantation	6/244 (2.5)	4/122 (3.3)	2/122 (1.6)	0.4
Neurological events				
Type 1	2/244 (0.8)	1/122 (0.8)	1/122 (0.8)	
Type 2	2/244 (0.8)	1/122 (0.8)	1/122 (0.8)	0.8
Type 3	1/244 (0.4)	0/122 (0)	1/122 (0.8)	
Bleeding requiring surgical revision	7/244 (2.9)	5/122 (2)	2/122 (0.8)	0.223
NYHA class at discharge				
I	215/230 (93.5)	115/122 (94.3)	100/108 (92.6)	

II	12/230 (5.2)	7/122 (5.7)	5/108 (4.6)	0.189
III				
IV	3/230 (1.3)	0/122 (0)	3/108 (2.8)	
NYHA III/IV	3/230 (1.3)	0/122 (0)	3/108 (2.8)	0.102
Medical treatment at discharge				
Vitamin k-antagonists/NOAC	225/231 (97.4)	109/109 (100)	116/122 (95.1)	*0.031*
ASA	87/230 (37.8)	26/108 (24.1)	61/122 (50.0)	*<0.001*
Dual antiplatelet therapy	2/230 (0.9)	2/108 (1.9)	0/122 (0)	0.219

Legend: AVR, aortic valve replacement; CABG, coronary artery bypass graft
